# Aliskiren for heart failure: a systematic review and meta-analysis of randomized controlled trials

**DOI:** 10.18632/oncotarget.21112

**Published:** 2017-09-21

**Authors:** Hongzhi Liu, Hongxing Luo, Suqin Wang, Cong Zhang, Jialiang Hao, Chuanyu Gao

**Affiliations:** ^1^ Department of Cardiology, Zhengzhou University People's Hospital, Zhengzhou, Henan, 450003, China

**Keywords:** aliskiren, heart failure, mortality, meta-analysis, systematic review

## Abstract

**Objective:**

To systematically review and synthesize the currently available evidence of aliskiren for the treatment of heart failure.

**Materials and Methods:**

We systematically searched the Cochrane, Embase and PubMed databases to identify the randomized controlled trials (RCT) on the effects of aliskiren on heart failure. Data were synthesized with random effects model and presented in forest plot. Publication bias was evaluated with funnel plot. Heterogeneity was evaluated with Begg's test and Egger's test.

**Results:**

Of 124 studies, 6 RCT of 9845 heart failure patients were included for meta-analysis, including 3727 patients receiving aliskiren. Compared with the controls, aliskiren did not significantly reduce the all-cause mortality (1.02 [0.91–1.14], I^2^ = 0%) or cardiovascular mortality (1.02 [0.88–1.17], I^2^ = 7.3%) of heart failure patients. Total adverse events, renal dysfunction, hypotension and hyperkalaemia were not significantly different between the aliskiren group and control group. Begg's test and Egger's test indicated low heterogeneity. Funnel plots indicated low publication bias.

**Conclusions:**

Aliskiren, either used alone or combined with standard medical therapy, does not significantly reduce the all-cause mortality or cardiovascular mortality of heart failure patients. Although aliskiren does not cause statistically higher adverse events, its adverse events may not be neglected.

## INTRODUCTION

Renin-angiotensin-aldosterone system (RAAS) activation plays a crucial role in the pathophysiologic changes of heart failure [1]. The deleterious effects of sustained RAAS activation on the development of heart failure are counteracted by angiotensin-converting enzyme (ACE) inhibitors, angiotensin II receptor blocker (ARB), and mineralocorticoid-receptor antagonist (MRA) [2–4]. Theoretically, renin inhibition is likely to have favourable therapeutic effects on RAAS activation since it not only blocks renin activation but also angiotensin and aldosterone activations. Aliskiren is a nonpeptide, orally active direct renin inhibitor which shows promising effects on blocking RAAS activation in animal studies [4]. However, some recent trials have demonstrated that it does not significantly improve the heart failure patients’ prognosis when used alone or combined with ACE inhibitors [5, 6].

Since there are no studies synthesizing the effects of aliskiren on heart failure patients, we aim to systematically review the previous studies on this topic and to analyse the adverse events associated with the use of aliskiren.

## RESULTS

**Figure 1:**
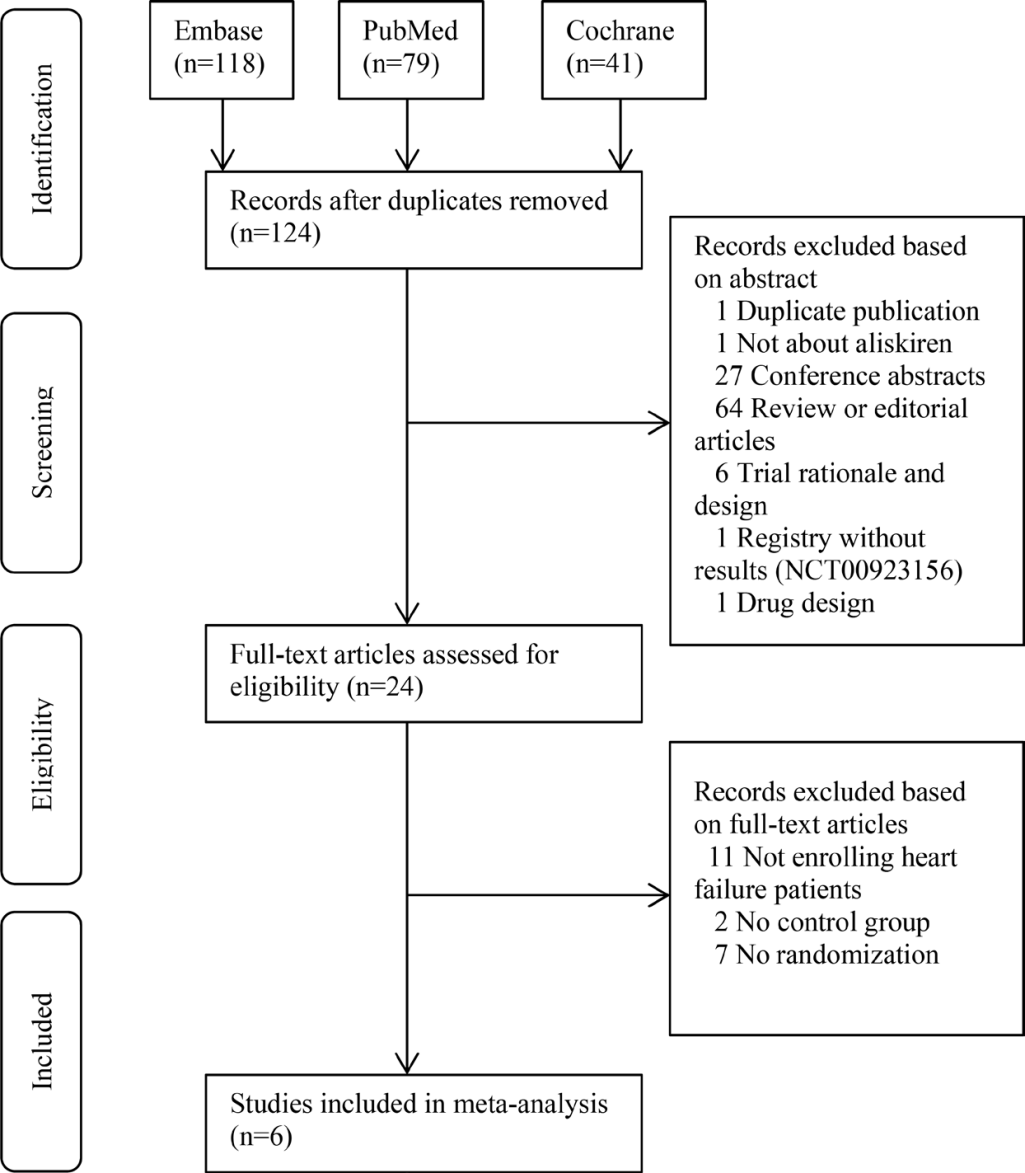
PRISMA flowchart of study selection

Figure 1 shows the PRISMA flow chart of this meta-analysis [8]. We primarily retrieved 118, 79 and 41 records from the Embase, PubMed and Cochrane Library, respectively. After removal of duplicate records, 124 studies were screened based on the abstracts and full-texts. For the first-round screening, irrelevant records including review, editorial and trial design were excluded. For the second-round screening, 24 records were evaluated. Then 11 records not enrolling heart failure patients, 2 not having control group and 7 not performing randomization were excluded. Six RCTs were finally included for meta-analysis [5, 6, 9–12].

### Baseline characteristics

Table 1 summarizes the basic characteristics of the eligible studies. A total of 9845 heart failure patients were enrolled in the eligible studies, with 3727 patients randomized to aliskiren group. Five studies investigated the effects of aliskiren on chronic heart failure and one on acute heart failure. The included patients were predominantly males with an advanced age and relatively low ejection fraction. Comorbidities including hypertension, diabetes mellitus and coronary artery disease were prevalent in the eligible heart failure patients. Most patients took various medications including ACE inhibitor, diuretic and beta-blocker. Of note, a very high proportion of patients (84–100%) took ACE inhibitors.

**Table 1:** Summary of basic characteristics of eligible studies

Author, year			Size	Age, (yrs)	Male, %	EF, (%)	NP, (pg/ml)	eGFR, (ml/min/1.73m2)	Hypertension, %	Diabetes, %	CAD/MI, %	ACEI/ARB, %	Diuretic, %	β-blocker, %	Follow-up, (m)
Seed A, 2007 [17]	–	CHF	27	64	89	25	198^*^	–	–	–	–	100	74	93	1 w
McMurray J, 2008 [11]	ALOFT	CHF	302	68	78	31	291^*^	69	100	30	68	99	–	94	3
Solomon SD, 2011 [18]	ASPIRE	CHF	820	60	83	38	–	80	53	23	20	100	–	96	9
Gheorghiade M, 2013 [5]	ASTRONAUT	AHF	1639	65	77	28	447^*^	67	76	41	55	84	96	83	11
Schroten NF, 2015 [19]	ARIANA-CHF-RD	CHF	41	68	82	34	932	49	41	26	62	100	90	90	6
McMurray J, 2016 [6]	ATMOSPHERE	CHF	7016	63	78	28	1194	74	62	28	41	–	80	92	37

EF = ejection fraction; NP = natriuretic peptide; eGFR = estimated glomerular filtration rate; CAD = coronary artery disease; MI = myocardial infarction; ACEI = angiotensin-converting enzyme inhibitor; AHF = acute heart failure; CHF = chronic heart failure; ALOFT = Aliskiren Observation of Heart failure Treatment; ASPIRE = Aliskiren Study in Post-MI Patients to Reduce Remodeling; ASTRONAUT = Aliskiren Trial on Acute Heart failure Outcomes; ARIANA-CHF-RD = Additive Renin Inhibition with Aliskiren on RBF and Neurohormonal Activation in patients with Chronic HF and Renal Dysfunction; ATMOSPHERE = Aliskiren Trial to Minimize Outcomes in Patients with Heart failure.

^*^ Indicates brain natriuretic peptide level. # Continuous data are expressed as mean or median.

### Mortality data

**Figure 2:**
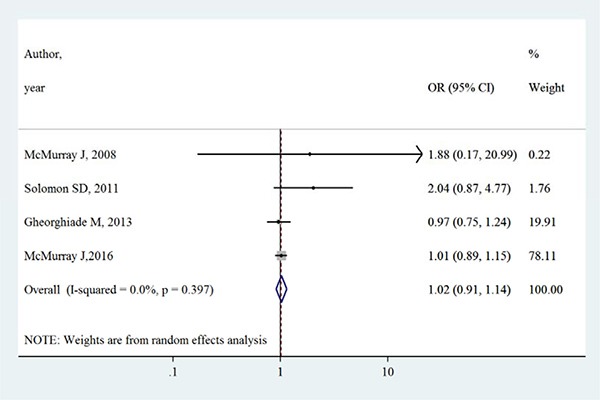
Effects of aliskiren on all-cause mortality in randomized controlled trials of heart failure with reduced ejection fraction patients

**Figure 3:**
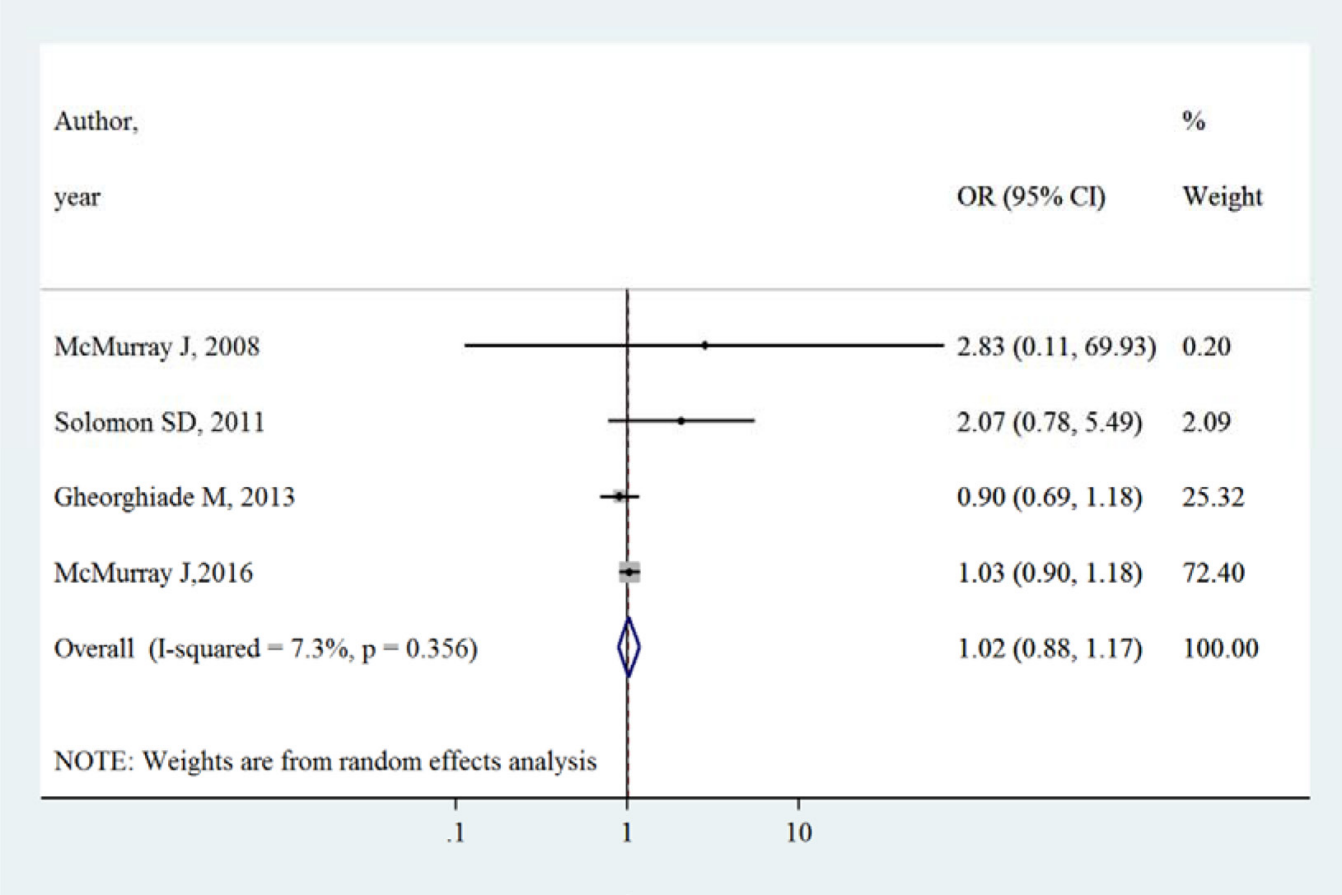
Effects of aliskiren on cardiovascular mortality in randomized controlled trials of heart failure with reduced ejection fraction patients

Table 2 summarizes the primary outcomes and secondary outcomes in intervention group and control group. The all-cause mortality varied between 1.3% −27.9% in intervention group, whilst between 0.7%–27.7% in control group. Both all-cause mortality and cardiovascular mortality were slightly higher in intervention group than control group. However, Figures 2 and 3 show that aliskiren is not significantly associated with lower all-cause mortality (OR [95% CI]:1.02 [0.91–1.14], I^2^ = 0%, *P* > 0.05) or cardiovascular mortality (OR [95% CI]:1.02 [0.88–1.17], I^2^ = 7.3%, *P* > 0.05). The I^2^ values of both outcomes were small, indicating low risks of inter-study heterogeneity.

**Table 2:** Summary of primary outcomes and secondary outcomes after receiving aliskiren

Author, year	All-cause mortality		Cardiovascular mortality	
	Intervention	Control	Intervention	Control
McMurray J, 2008 [11]	2/156	1/146	1/156	0
Solomon SD, 2011 [12]	17/423	8/397	13/423	6/397
Gheorghiade M, 2013 [5]	144/808	148/807	126/808	137/807
McMurray J, 2016 [6]	654/2340	646/2336	562/2340	547/2336

### Adverse events

**Figure 4:**
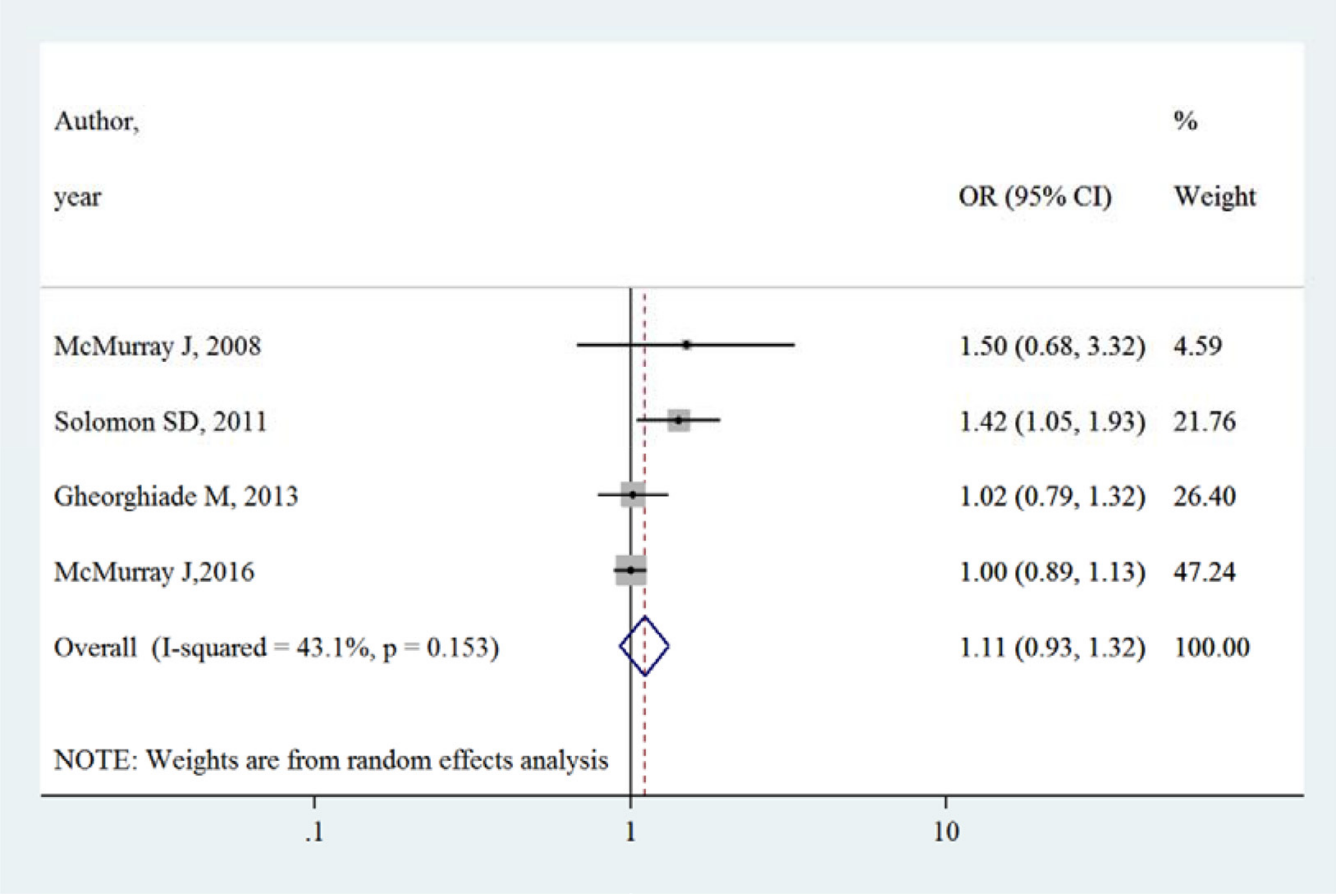
Effects of aliskiren on total adverse events in randomized controlled trials of heart failure with reduced ejection fraction patients

**Figure 5:**
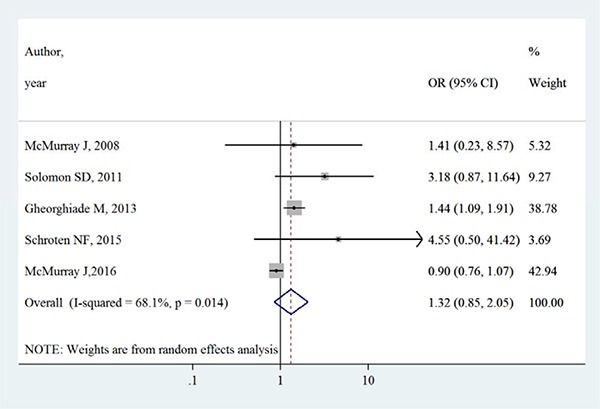
Effects of aliskiren on renal dysfunction in randomized controlled trials of heart failure with reduced ejection fraction patients

**Figure 6:**
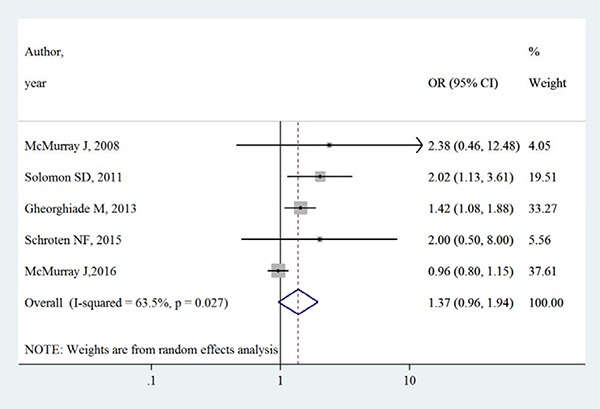
Effects of aliskiren on hypotension in randomized controlled trials of heart failure with reduced ejection fraction patients

**Figure 7:**
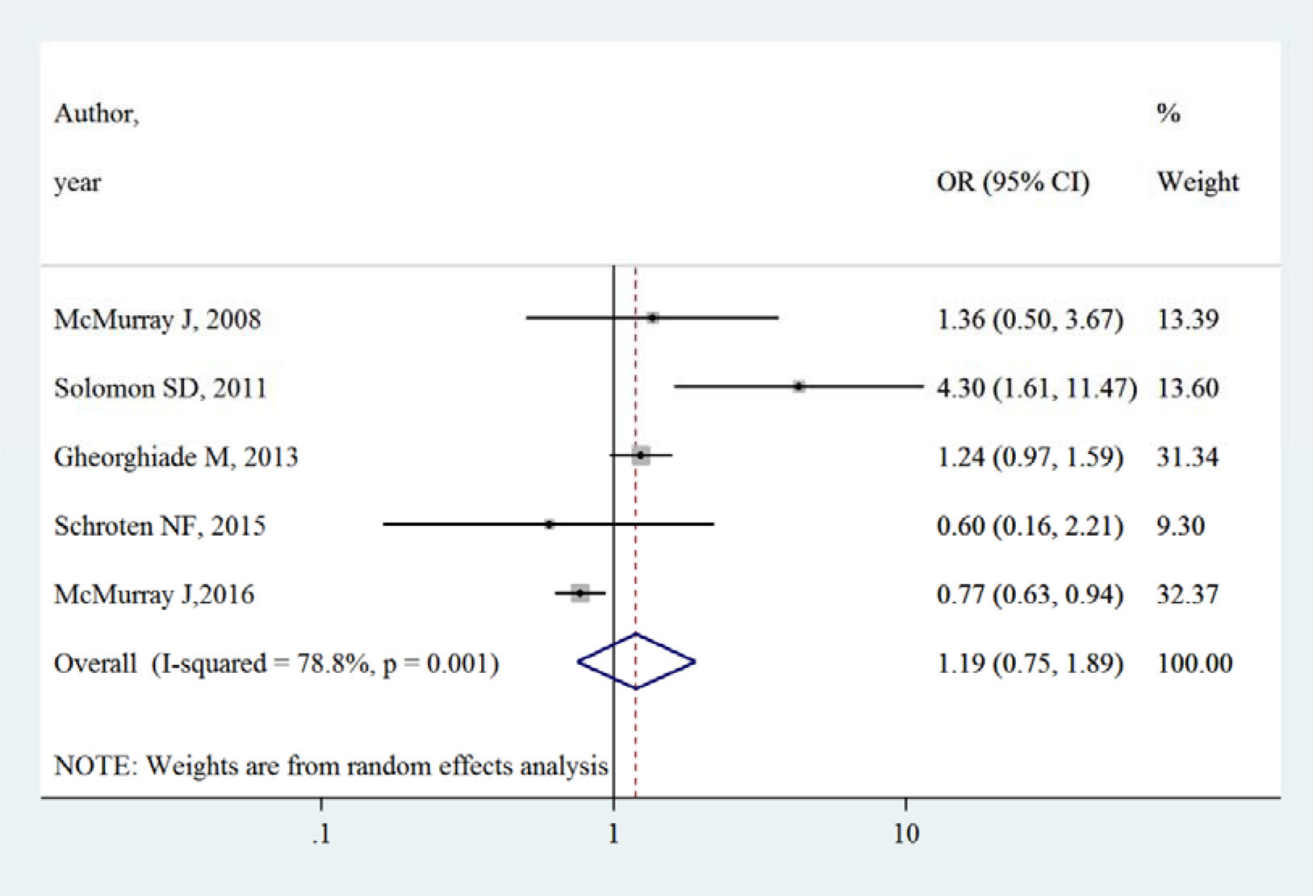
Effects of aliskiren on hyperkalaemia in randomized controlled trials of heart failure with reduced ejection fraction patients

Table 3 summarizes the occurrence of adverse events in aliskiren group and control group. Total adverse events, renal dysfunction, hypotension and hyperkalaemia were higher in intervention group in most adverse events, except that in the ATMOSPHERE trial (Aliskiren Trial to Minimize Outcomes in Patients with Heart failure), more renal dysfunction and hyperkalaemia events were reported in control group. However, Figures 4, 5, 6 and 7 show that total adverse events (OR [95% CI]:1.11 [0.93–1.32], I^2^ = 43.1%), renal dysfunction (OR [95% CI]:1.32 [0.85–2.05], I^2^ = 68.1%), hypotension (OR [95% CI]:1.37 [0.96–1.94], I^2^ = 63.5%) and hyperkalaemia (OR [95% CI]:1.19 [0.75–1.89], I^2^ = 78.8%) were not significantly different between intervention group and control group (all *P* > 0.05).

**Table 3:** Summary of adverse events

Author, year	Total adverse events		Renal dysfunction		Hypotension		Hyperkalemia	
	Int	Con	Int	Con	Int	Con	Int	Con
McMurray J, 2008 [11]	17	11	3	2	5	2	10	7
Solomon SD, 2011 [12]	316	268	10	3	37	18	22	5
Gheorghiade M, 2013 [5]	670	667	134	98	138	102	169	142
Schroten NF, 2015 [10]	–	–	7	1	12	4	12	8
McMurray J, 2016 [6]	1504	1501	279	306	249	258	192	243
								

### Publication bias and small-study effects

**Figure 8:**
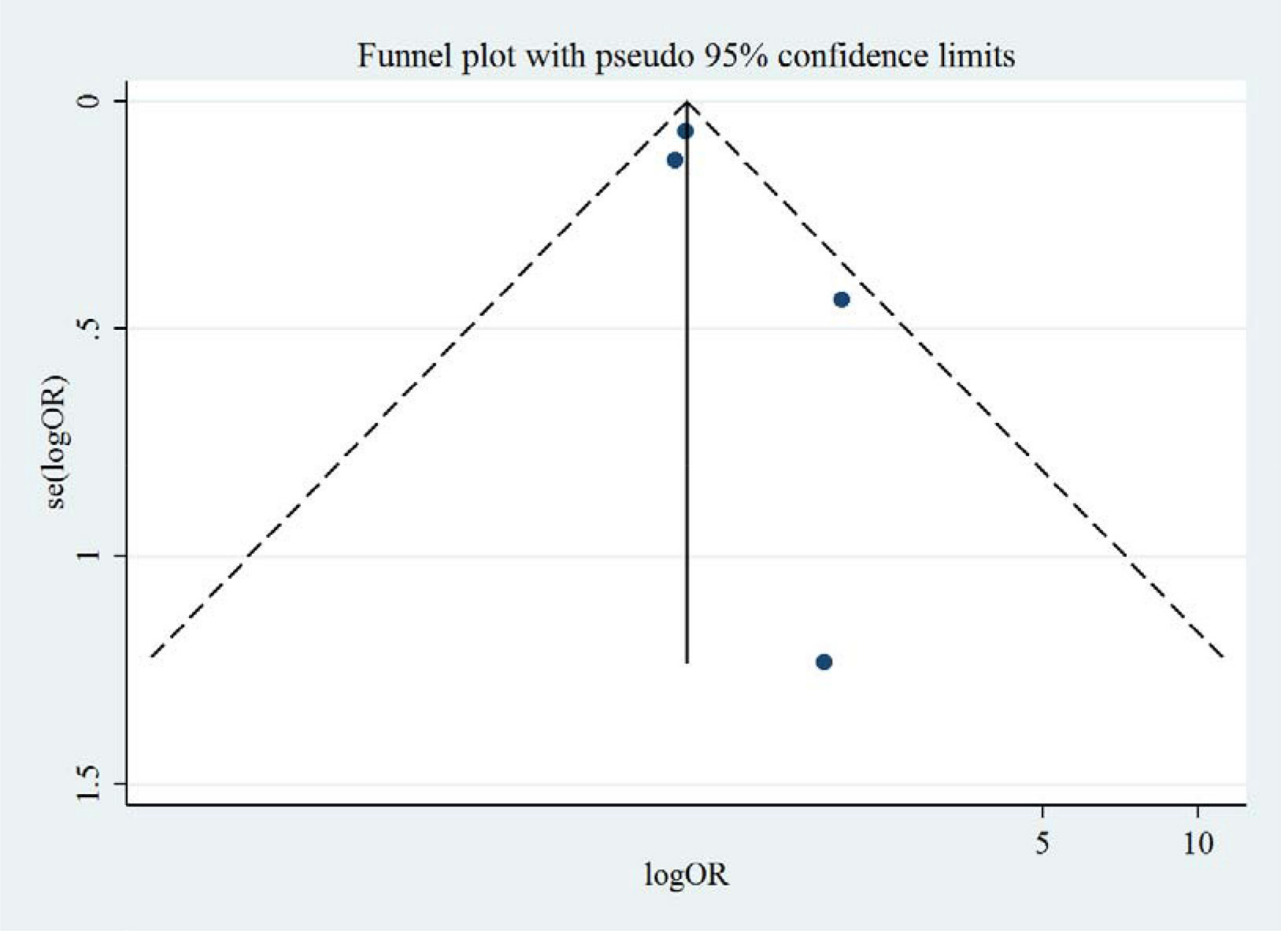
Funnel plot of aliskiren on all-cause mortality in randomized controlled trials of heart failure with reduced ejection fraction patients

**Figure 9:**
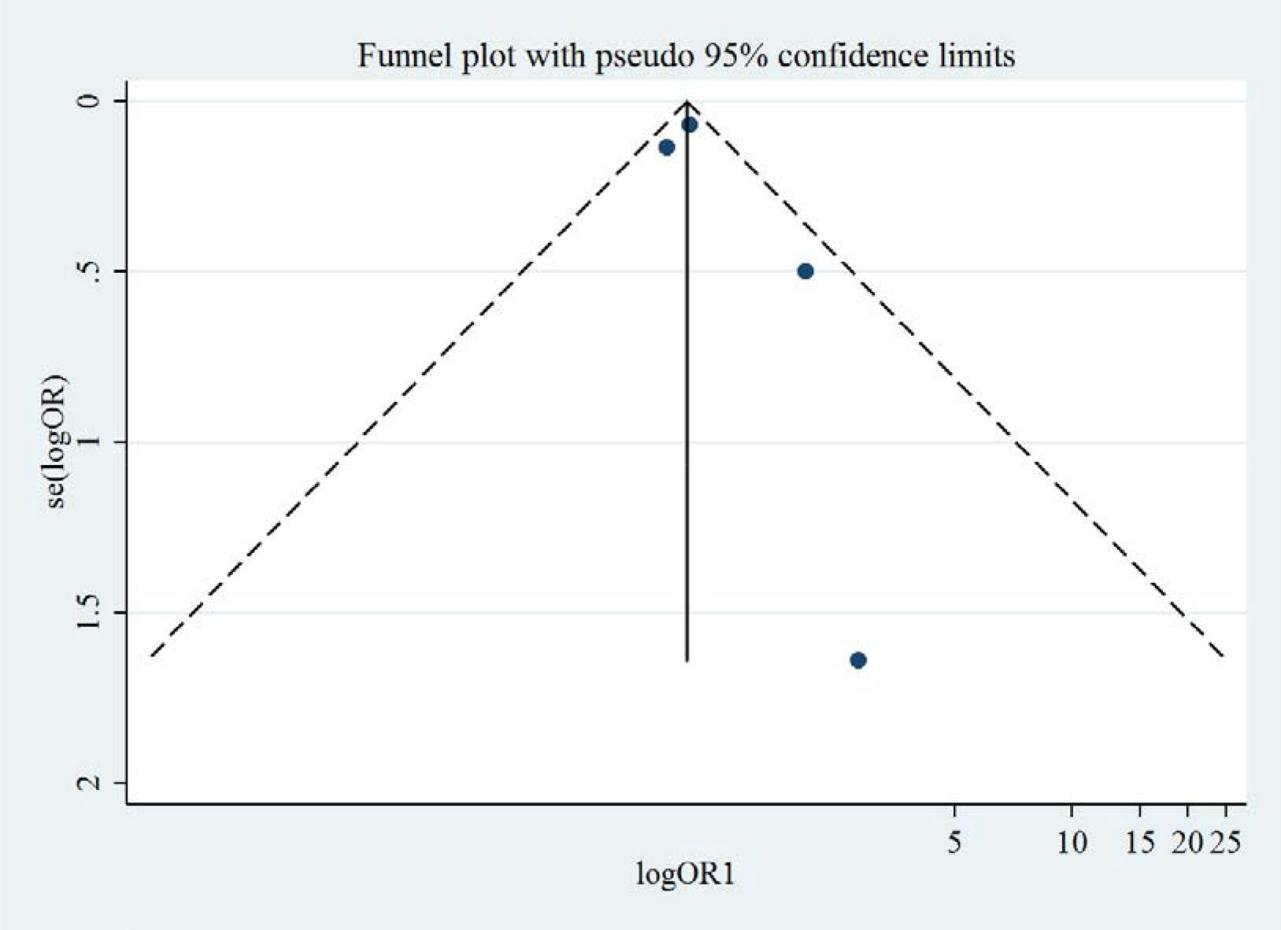
Funnel plot of aliskiren on cardiovascular mortality in randomized controlled trials of heart failure with reduced ejection fraction patients

Figures 8 and 9 show the symmetric funnel plots of all-cause mortality and cardiovascular mortality, without any studies falling out of the funnels, indicating low risks of publication bias. Begg's test and Egger's test did not indicate any small-study effects for all-cause mortality or cardiovascular mortality (all *P* > 0.05).

## DISCUSSION

To the best of our knowledge, this is the first meta-analysis of aliskiren for heart failure patients. Our results show that aliskiren is not superior to placebo in reducing all-cause mortality or cardiovascular mortality of heart failure patients. Adverse events are slightly higher in aliskiren group, but the differences have not reached statistical significance. Although RAAS activation is a compensatory action to the decreased cardiac output in heart failure patients and the blockage of RAAS with the drugs such as ACE inhibitor, ARB and MRA have yielded great progresses in the last decades; the direct inhibition of renin does not bring benefits to the heart failure patients who have received optimal medications. Renin, a 340-amino acid protease polypeptide secreted from renal juxtaglomerular apparatus to blood circulation, serves as the first rate-limiting substance of RAAS [13]. It is also the first orally active nonpeptide with a plasma half-life of about 24 hours [9]. It was once supposed to be very powerful in reducing the heart failure patients’ mortality, but the results of RCT show that it fails to reach such a high goal.

Our findings do not support the role of direct renin inhibitor aliskiren as a favourable drug for heart failure patients who have received optimal medications, although some studies do show that it decreases plasma renin concentration and activity. Aliskiren has also been reported to not decrease the mortality and adverse events of patients with myocardial infarction, prehypertension, or type 2 diabetes mellitus [14–16]. Aliskiren, either used alone or combined with standard medical therapies containing ACE inhibitor or ARB, is associated with more adverse events including hypotension, renal dysfunction, and hyperkalaemia in heart failure patients [6]. These adverse events such as hypotension seem to be the side effects of excessive inhibition of RAAS. It appears that there is an unknown upper limit for the benefits of RAAS blockade [17]. In other words, it's critical to know whether or not there is an optimal inhibition of renin level so as to achieve the best outcomes for heart failure patients. Another perplexing observation is that although aliskiren decreases the natriuretic peptides level of heart failure patients, it does not reduce the all-cause mortality or cardiovascular mortality. Both the Aliskiren Observation of Heart failure Treatment (ALOFT) study and the Aliskiren Trial on Acute Heart failure Outcomes (ASTRONAUT) study have showed that aliskiren significantly decreased NT-proBNP and BNP levels, but the patients’ outcomes were not improved. The results are contradictory to the conventional view on natriuretic peptides which have been widely recognized as one of the most powerful predictors of the prognosis of heart failure patients [9].

The failure of aliskiren has brought to us some implications for future development of RAAS inhibitors. Firstly, a RAAS inhibitor may not suit all heart failure patients. Since aliskiren has been associated with adverse events such as hypotension and hyperkalaemia, it may be best indicated for the heart failure patients who have a past history of hypertension and laboratory examination of hypokalaemia [18]. Most studies of our meta-analysis have included heart failure with reduced ejection patients. Nevertheless, a recently published small-scale RCT has preliminarily shown that aliskiren reduce arterial stiffness and left ventricular diastolic function in elderly hypertensive patients during a follow-up of 6 month [19]. The study indicates that aliskiren is possible to be effective for heart failure with preserved ejection fraction patients, rather than those with reduced ejection fraction. In other words, drug designers should bear in minds that a RAAS inhibitor may be pharmacologically effective for a small proportion of heart failure patients, while trial designers should remember that the inclusion criteria of a RCT should be very carefully designed so that it best covers the most indicated patients. Secondly, the inhibition of RAAS may suffer from ceiling effects. The direct inhibition of renin is supposed to bring with the most effective inhibition of the RAAS. In fact, the renin concentration and activity do decrease following the use of aliskiren, but this kind of inhibition is seemingly excessive. Maybe the question that we should keep asking ourselves is that what is the best level of controlling renin and RAAS system, so as to improve the prognosis of heart failure patients to the maximum extent? Thirdly, natriuretic peptides including BNP and NT-proBNP have been extensively recognized as the most important prognostic factor of heart failure patients, but the studies of aliskiren have warned us that this kind of reduction of natriuretic peptides may not be associated with better outcomes in heart failure patients. Heart failure may be a syndrome that requires a comprehensive management strategy rather than relying on a simple laboratory indicator.

Our study is limited by some factors. Firstly, we restricted our publication language to English, so the studies published in other languages were not included in our meta-analysis. However, we believe that the addition of extra studies into our meta-analysis is unlikely to utterly change our conclusion because the ATMOSPHERE study has the largest sample size of 7016, which is unlikely to be surpassed by any other studies the future. Secondly, we identified moderate to high heterogeneity in some adverse events such as renal dysfunction, hypotension and hyperkalaemia, probably attributable to the comparatively low event rates in both intervention group and control group.

In conclusion, our results show that currently available randomized controlled trials do not support a beneficial role of aliskiren in improving the prognosis of heart failure patients. Future clinical trials of RAAS inhibition, especially renin inhibition, may continue to improve the heart failure patients’ outcomes but they should also be preceded with caution so as to avoid potential adverse events on the patients.

## MATERIALS AND METHODS

### Definition and search strategy

In this systematic review, heart failure was defined with the following criteria 1) symptoms and/or signs; 2) decreased left ventricular ejection fraction and/or natriuretic peptides; 3) echocardiographic evidence of systolic or diastolic dysfunction. The primary outcomes of this study were all-cause mortality and cardiovascular mortality. We also collected the adverse events reported in the eligible studies. We identified observational and interventional studies published in Cochrane, Embase and PubMed databases until October 25, 2016 with various combinations of “aliskiren” and “heart failure”. We limited our search to English publications.

### Inclusion and exclusion criteria

A study was included if it 1) included heart failure patients 2) administered aliskiren to the intervention group, 3) had a control group, 4) reported all-cause mortality, cardiovascular mortality or adverse events. Exclusion criteria were as follows: 1) not enrolling heart failure patients, 2) conference abstract, 3) review or editorial articles, 4) trial rationale and design, 5) no control group, 6) not reporting outcome data.

### Data extraction

Data were independently extracted by 2 reviewers (HXL and HZL) using a standardized data extraction form. Discrepancies were resolved by consensus. The following basic characteristics of eligible studies were included: first author, publication year, sample size, age, sex, left ventricular ejection fraction (LVEF), natriuretic peptide level, estimated glomerular filtration rate (eGFR), comorbidities, medication use and follow-up period. All-cause mortality, cardiovascular mortality and adverse events were extracted for meta-analysis.

### Statistical analysis

Odds ratios (OR) and the corresponding 95% confidence intervals (CI) were calculated for outcomes and adverse events in this study. Heterogeneity across trials was evaluated with Q-statistic and I^2^-statistic [7]. I^2^ represents the percentage of total variation across studies resulting from heterogeneity rather than chance. A value of 0% indicates no heterogeneity, with larger values indicate higher heterogeneity. Begg's test and Egger's test were performed to identify small-study effects. Publication bias was visually estimated by a funnel plot. All analyses were performed with Stata 14.0 (Stata, College Station, Texas, USA).
